# Chemically synthesized zinc finger molecules as nano-addressable probes for double-stranded DNAs

**DOI:** 10.1186/1477-3155-3-5

**Published:** 2005-06-29

**Authors:** Markus von Nickisch-Rosenegk, Eva Ehrentreich-Forster, Rothin Strehlow, Alexander Christmann, Frank F Bier

**Affiliations:** 1 Fraunhofer Institute of Biomedical Engineering (IBMT), Dpt. Molecular Bioanalytics and Bioelectronics, Arthur-Scheunert-Allee 114-116, 14558 Nuthetal, Germany

## Abstract

Our experiments describe an alternative method of dsDNA recognition using zinc finger (ZF) molecules which bind DNA specifically and with high affinity. Our aim was to develop zinc finger probes which are able to bind to dsDNA molecules at predetermined sites. In our basic approach we used pairs of complementary oligonucleotides to form dsDNAs, containing one of the three SP1-transcription factor motifs as a zinc finger recognition site. Two zinc finger probes of the SP1 motif were chemically synthesized and modified with a Dy-633 fluorophore. The SP1 peptides were folded into functional zinc fingers using zinc chloride. The addressable dsDNAs were immobilized on optical fibres, and the kinetics and binding rates of the artificial zinc finger probes were measured by a fluorescence detecting device (photomultiplying tube). The two zinc fingers and their corresponding DNA recognition sites served as specific probes and controls for the matching site and vice versa. Our experiments showed that a variety of dsDNA-binding probes may be created by modification of the amino acid sequence of natural zinc finger proteins. Our findings offer an alternative approach of addressing dsDNA molecules, for example for use in a nanoarray device.

## Background

Nanostructures based on DNA have been reported previously. Generally it is a prerequisite to generate single stranded DNA (ssDNA) molecules to open the possibility of probing with oligonucleotides. However, the production of long ssDNA may not be an easy task. ssDNAs are more sensitive to physical handling, tend to form stable secondary structures and require considerable effort to obtain sufficient quantities intact molecules. An alternative approach relies on the use of peptide-nucleic acids (PNAs), which are capable of recognizing dsDNAs. The recent reports have demonstrated that this approach is not always practical and PNA binding is not always reliable [1]. Another potential approach is to use existing cellular mechanisms involved in the control of the production of proteins on the level of nucleic acids. Transcription factors which bind to dsDNA target sequences are switching specific genes "on" or "off" by recognizing and binding to short specific DNA fragments. Zinc finger proteins are transcription factors which have concise and simple structure [2], can be made synthetically and might therefore provide alternative probes for dsDNA recognition [3].

## Results and discussion

**Figure 1:**
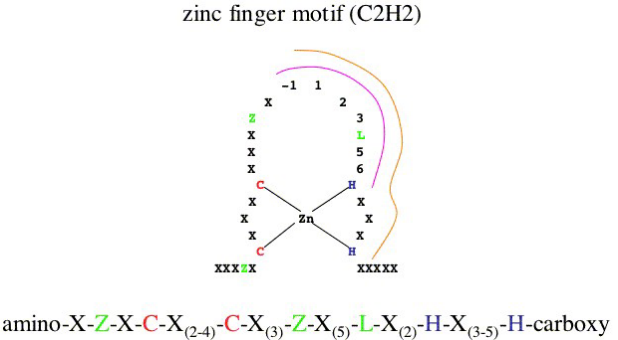
Scheme of a C2H2 zinc finger motif. X denotes any amino acid; Z – hydrophobic amino acids [L, I, V, M, F, Y, W, C]; L is Leucine; C is Cysteine; H is Histidine. The binding interface of the **α-Helix **is formed by amino acids No -1, 1, 2, 3, L, 5 and 6.

In our experiments we used one of the three zinc finger motifs of the SP1-zinc-finger as a template for our chemically synthesized zinc finger probes, which were of the C2H2 type (Figure 1). The DNA-binding region was altered in two places to enable the folded zinc finger to bind either at the "AAA"-DNA sequence site or at the "GCG"-DNA sequence site (see M & M, bold and underlined). The custom-made peptides were modified with a fluorophore (Dye633) at their amino-termini to allow quantitative detection. The double-stranded DNA targets were made of custom-made complementary oligonucleotides carrying the appropriate DNA recognition-sequence and a phosphate modification at the 5'-end of one of the complementary oligonucleotides for their immobilization.

**Figure 2:**
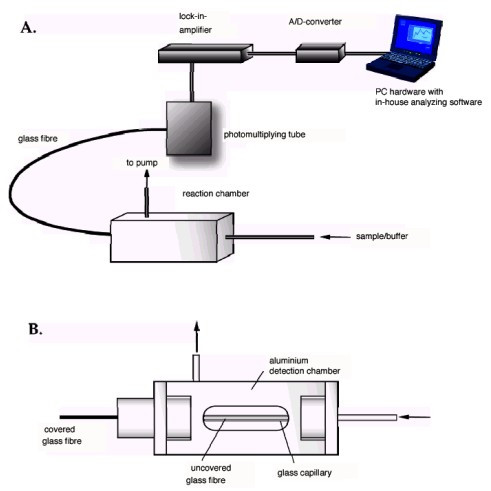
Scheme of the used fibre optical device. A) complete work flow. B) Reaction and detection chamber (cross section) (derived from Schwonbeck 2004)

**Figure 3:**
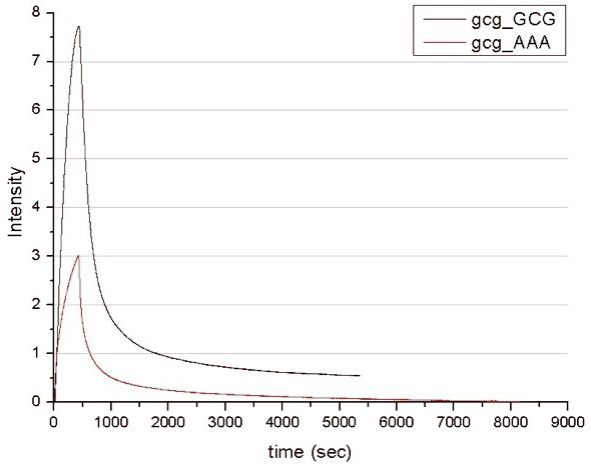
Real-time detection of the binding kinetics to the "GCG"-sequence immobilised on a glass fibre. Black curve corresponds to the interaction between matching pair of ZF1 and "GCG" motif (k_D_: 4,9·10^-3 ^/s), the red curve corresponds to the ZF2 (k_D_: 7,3·10^-3 ^/s) which should recognise the "AAA" sequence, but not the "GCG".

**Figure 4:**
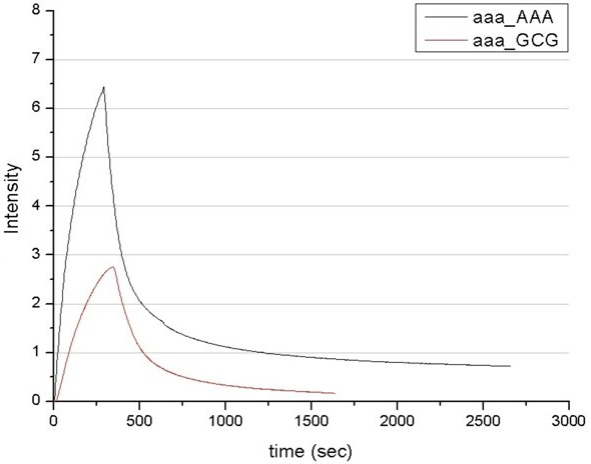
Real-time detection of the binding kinetics to the "AAA"-sequence immobilised on a glass fibre. Black curve corresponds to the interaction between matching pair of ZF2 and "AAA" motif (k_D_: 3,5·10^-3 ^/s), the red curve corresponds to the ZF1 (k_D_: 4,6·10^-3 ^/s) which should recognise the "GCG" sequence, but not the "AAA".

Detection was done by fluorescence on a fibre-optical-device developed in our institute (Figure 2), which was driven by the in-house built software. The double-stranded DNA targets were immobilized on a silanized glass fibre and incubated with the zinc fingers in a flow-through chamber (Figure 2). The binding and dissociation events were measured in real time and binding curves (binding signal vs. time) were obtained (Figures 3 and 4). The curves were fitted using in-house built fitting routine. The resulting dissociation rate (k_D_) in a millimolar range were obtained, indicating high rates of clearance of the zinc finger molecules (see Figures 3 and 4). However, these were sufficient for our experiments. We have measured for the first time the specific binding of synthetic single-motif zinc finger peptides to their specific binding sites in real time *in vitro*.

Figure 3 shows a typical result obtained using the fibre covered with DNA containing the "GCG" motif (the motif recognised by ZF1). The black curve represents ZF1 binding, which is characterised by fast association kinetics. A small amount of ZF1 remained associated with the DNA for over 1.5 hours into the dissociation stage. A subsequent incubation of the same fibre with ZF2 (Figure 3, red curve) showed faster dissociation kinetics. Similarly, the ZF2 motif bound the "AAA" sequence strongly and significant amount of ZF2 remained bound to the DNA irreversibly (Figure 4, black curve), whereas ZF1 dissociated completely (Figure 4, red curve).

## Conclusion

In this work we have shown that synthetic zinc fingers bind specifically to their binding sites which consist of nucleic acid triplets. An additional advantage of the SP1 system is that it offers an opportunity to use multiple single-motif zinc fingers [4,5], similar to the native SP1 transcription factor motifs. Using multiple ZF domains should result in binding affinities in a nanomolar range and should also allow for more specific sequence recognition. We envisage that nano-addressable zinc finger motif-based probes for dsDNAs will employ multiple linked ZF motifs and possess binding affinities in the nanomolar range.

## Materials and methods

### Nucleic acids

Complementary oligonucleotides (Carl Roth, Karlsruhe) with specific DNA-zinc-finger-recognition sites were:

### GCG-motif

5' -Phosphat- TgACTgACTgACTgACTgAC**gCg**TgACTgACTgAC -3'(sense)

5' -gTCAgTCAgTCA**CgC**gTCAgTCAgTCAgTCAgTCA-3'(antisense)

### AAA-motif

5' -Phosphat-TgACTgACTgACTgACTgAC**AAA**TgACTgACTgAC-3'(sense)

5' – gTCAgTCAgTCA**TTT**gTCAgTCAgTCAgTCAgTCA -3'(antisense)

Corresponding pairs of the sense and antisense oligonucleotides were annealed to yield dsDNA fragments, which were used for immobilisation (see below)

### Synthetic peptides (zinc fingers in "one-letter-code")

The custom-made peptides were modified with a fluorophore (Dye633, Dyomics) at the amino-terminus for detection. The peptides were ZF1 (GCG-motif): N-Dy633-GFMCTWSYCGKRFT**RSDELQR**HKRTH-COOH (Biosynthan, Berlin) and ZF2 (AAA-motif):

N-Dy633-GFMCTWSYCGKRFT**QSSNLQT**HKRTH-COOH (peptides & elephants, Nuthetal).

Storage buffer contained 10 mM Tris/HCl (pH 7,5), 90 mM KCl, 1 mM MgCl_2_, 90 μM ZnCl_2_, 5 mM DTT; running buffer was made by adding acetylated BSA(Aurion, Wageningen NL) to the storage buffer to a final concentration of 1–2%. The peptides were dissolved in the running buffer (at the concentration of 0.5 μg peptide / 500 μl buffer) and incubated for 15 min to allow peptides to fold to form zinc fingers prior to incubation with DNA (Association stage). To measure the dissociation kinetics, the buffer was changed to the "running" buffer (without ZF).

### Detection

Detection was using "in-house" built optical fibre device "Horst" (see Figure 2), driven by in-house software.

### Silanized glass fibres

Glass fibres were cleaned with freshly prepared hot "Piranha"-solution (1:2 mixture of 30% hydrogen peroxide and 97% sulphuric acid) for 1 minute and thoroughly rinsed with distilled water. The fibres were further incubated in freshly prepared hot 10N NaOH for 2 h. The activated fibres were immersed in 10% aminopropyl-triethoxysilane (APTES) solution, pH adjusted to 3.5 and heated to 80°C in a water bath for 2 h. Finally the fibres were rinsed with HPLC grade water again and baked at 110°C for 1 h. The amino-modified surface is stable for several months.

### Immobilization of DNA

A solution of 5'-phosphorylated oligonucleotide was diluted with 30 mM 1-methylimidazole to a final concentration of 5 μM. Then 10 mg/ml EDC was added to each oligonucleotide and mixed for 1 minute. The oligonucleotide solutions were used immediately to cover the activated parts of the glass fibres (see above).
